# Quantifying Competitive Exclusion and Competitive Release in Ecological Communities: A Conceptual Framework and a Case Study

**DOI:** 10.1371/journal.pone.0160798

**Published:** 2016-08-18

**Authors:** Hila Segre, Niv DeMalach, Zalmen Henkin, Ronen Kadmon

**Affiliations:** 1 Department of Ecology, Evolution and Behavior, The Hebrew University of Jerusalem, Givat Ram, Jerusalem, Israel; 2 Beef Cattle Section, Newe-Ya'ar Research Center, Department of Natural Resources, Agricultural Research Organization, Ramat Yishay, Israel; Chinese Academy of Forestry, CHINA

## Abstract

A fundamental notion in community ecology is that local species diversity reflects some balance between the contrasting forces of competitive exclusion and competitive release. Quantifying this balance is not trivial, and requires data on the magnitude of both processes in the same system, as well as appropriate methodology to integrate and interpret such data. Here we present a novel framework for empirical studies of the balance between competitive exclusion and competitive release and demonstrate its applicability using data from a Mediterranean annual grassland where grazing is a major mechanism of competitive release. Empirical data on the balance between competitive exclusion and competitive release are crucial for understanding observed patterns of variation in local species diversity and the proposed approach provides a simple framework for the collection, interpretation, and synthesis of such data.

## Introduction

Competitive exclusion and competitive release are two sides of the same coin: competitive exclusion refers to situations in which a species is excluded from a local community by competitive interactions with other species, while competitive release refers to situations in which a certain factor (hereafter, a 'releasing factor') limits the ability of the competitors to exclude a species, thereby allowing it to exist in the community. The hypothesis that species diversity reflects some balance between these mechanisms lies at the heart of classical ecological theory, and understanding the extent to which and the mechanisms by which these contrasting forces affect the diversity of ecological communities has been a central question in ecology [1–8].

**Fig 1:**
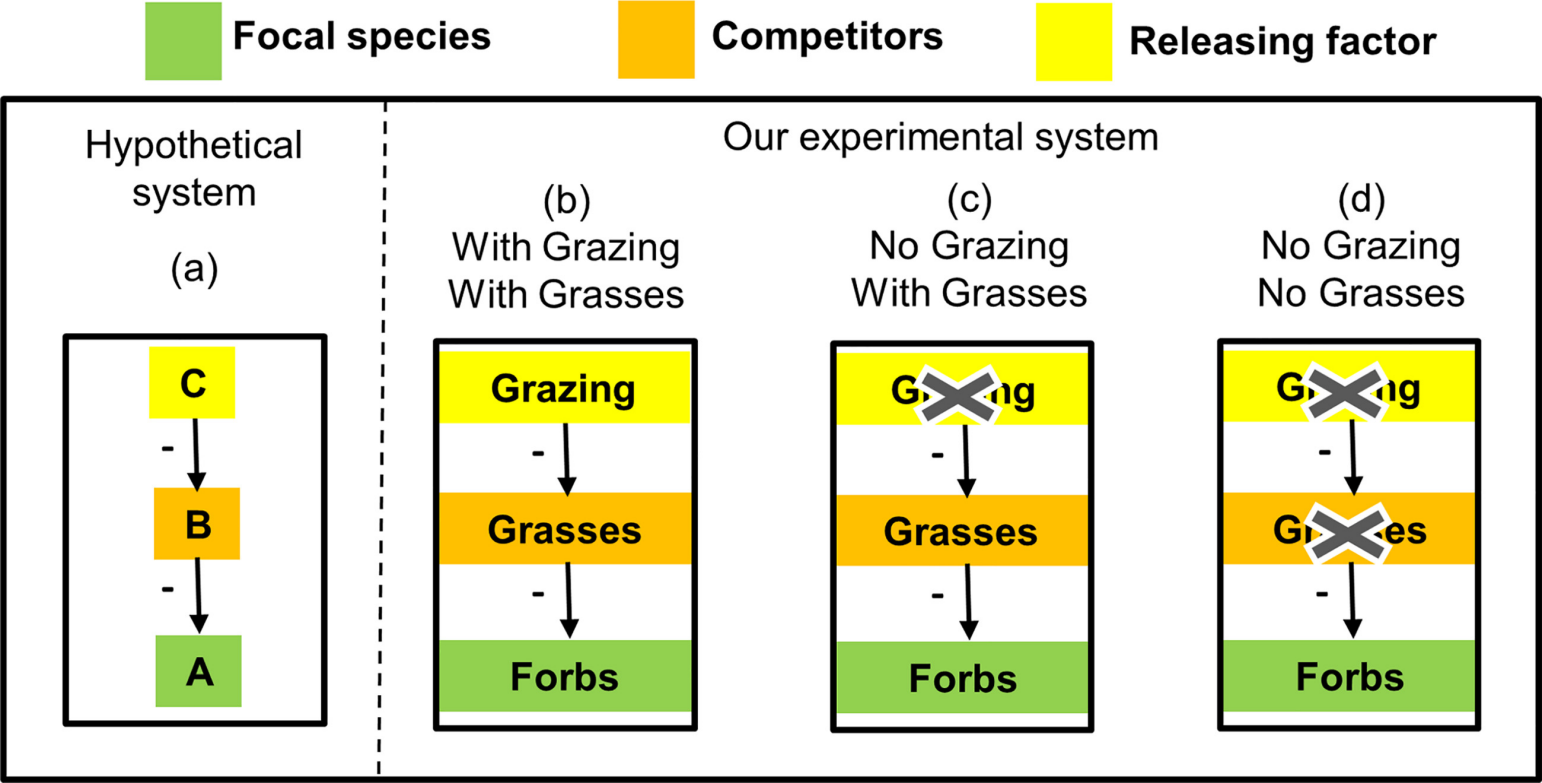
**A schematic presentation of a simple system of competitive release (a) and the design of our experiment (b-d).** In the most simple system (a), a releasing factor (C) allows a focal species (A) to persist in a community by releasing it from the negative effect of a superior competitor (B). In our experimental system (b) grazing is treated as the releasing factor, forbs as the focal species, and grasses as the superior competitors. The magnitude of competitive exclusion is quantified by comparing forb richness in plots with (c) and without (d) grasses in the absence of grazing, and the magnitude of competitive release is quantified by comparing plots with (b) and without (c) grazing.

The simplest system of competitive release consists of three elements–a focal species, a competitor capable of excluding the focal species from the community, and a releasing factor capable of preventing such competitive exclusion (Fig 1A). A classic example is the rocky intertidal system studied by Menge [9]. In this system, a predator snail (*Thais lapillus*) is capable of releasing a focal species (the barnacle *Balanus balanoides*) from the competitive effect of a superior competitor (the mussel *Mytilus edulis*). This system represents what Wootton [10] called an 'interaction chain': one species (the releasing factor) indirectly affects another (the focal species) by influencing the abundance of an intermediate species (the competitor) that interacts with both (Fig 1A). However, most natural systems are more complex, and may include a larger number of focal species and/or competitors, as well as more complex interactions. It is also important to note that competitive release differs from 'trophic cascades': although in both cases the releasing factor might be a predator, competitive release involves a maximum of two trophic levels and the 'release' is from competition, while a trophic cascade involves at least three trophic levels and the release is from predation.

Although competitive exclusion and competitive release are strongly linked to each other, empirical studies focusing on species diversity usually consider only one side of this game. As a result, in spite of a considerable progress in our understanding of the mechanisms by which potentially releasing factors such as predation, grazing, and disturbance, affect the diversity of ecological communities[5, 7, 11–16] little is known about the actual *effectiveness* of such factors in offsetting species loss caused by competitive exclusion.

In this contribution we attempt to reduce this gap by providing a conceptual framework for empirical studies of the *balance* between the effects of competitive exclusion and competitive release on species diversity. The proposed framework provides a simple measure for the effectiveness of competitive release in mitigating the negative effect of competitive exclusion on species diversity, and an experimental methodology for quantifying this measure. Our study has two parts. In the first part, we describe the proposed framework and in the second part we demonstrate its applicability with a case study focusing on an annual grassland system where grazing is hypothesized to be a major releasing factor.

Grasslands have been used extensively to developing and testing theories on both plant competition [17–22] and grazing [12, 13, 23–26]. There are also studies in which the combined effects of competition and grazing on plant species diversity were studied in the same system [23, 27]. However, as far as we are aware, the case study described here is the first attempt to explicitly quantify the balance between these two fundamental forces.

### A Conceptual Framework

Our framework is based on the concept of *'effectiveness'*, which we define as *the ability of a particular releasing factor to offset species loss caused by a particular competitor (or a set of competitors) in a particular system*. Thus, effectiveness is defined with respect to the effects of a specific releasing factor and a specific set of competitors in a specific system. It is calculated as the ratio between the number of species released from competitive exclusion by the relevant releasing factor, and the number of species that are competitively excluded by the relevant competitor(s) in the absence of the releasing factor. By using this definition we focus on competitive effects on species richness, and ignore possible effects on species composition.

**Fig 2:**
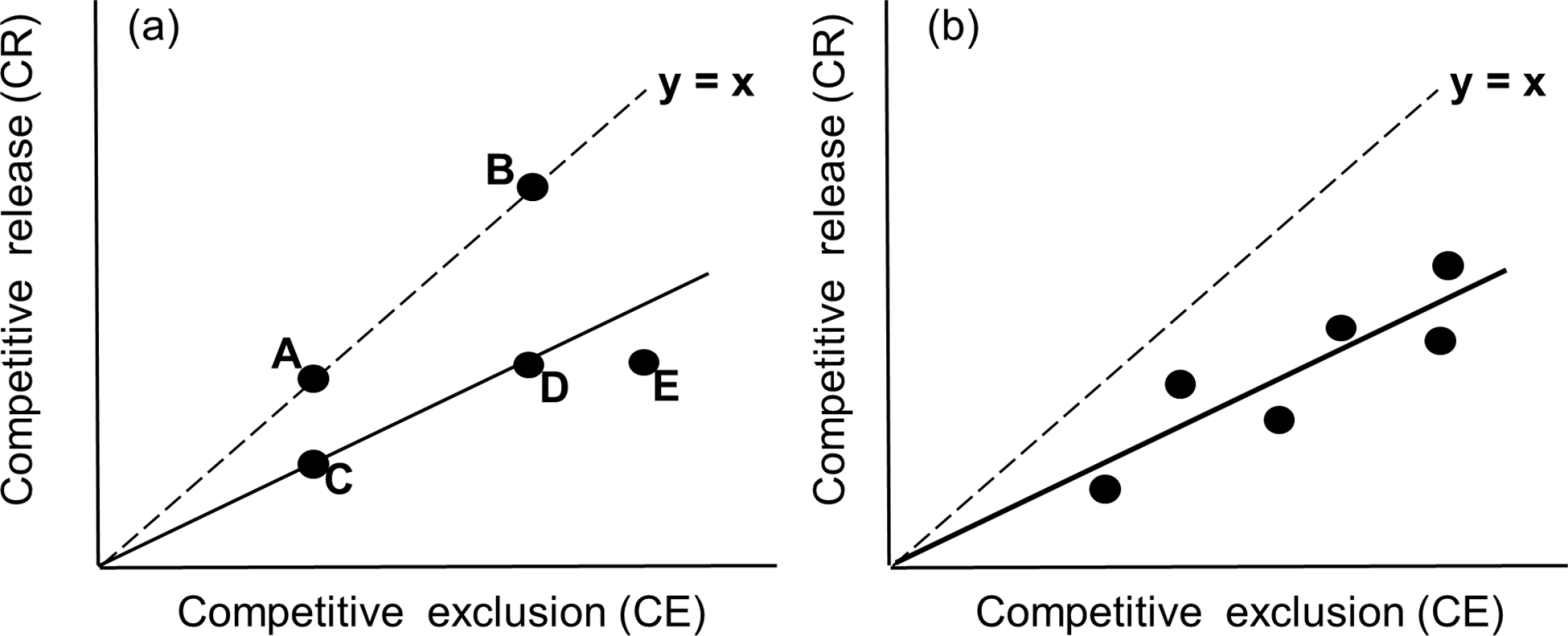
The CR-CE space as a framework for analyzing the balance between competitive release and competitive exclusion. Systems in which the releasing factor fully compensates for competitive exclusions fall on the line y = x (the 'compensation line', points A and B in (a)). Systems characterized by partial compensation fall below the compensation line (C, D, E in (a)). Note that points C and D have the same effectiveness although they differ in the magnitude of competitive exclusion. Point E shows a lower effectiveness than points C and D although the magnitude of competitive release is similar to point D. If data on both forces are available for a set of sites within the same system, the effectiveness of the releasing factor can be expressed by the slope of a linear regression fitted to the data (b).

The above measure of effectiveness can be quantified in the field using a 'two-sided' experiment that combines removal of the releasing factor (to determine its contribution to species diversity) and removal of both the releasing factor *and* the competitors (to determine the magnitude of competitive exclusion in the absence of the releasing factor). Assuming that both experiments are conducted in a given site, we can present their results as a single point in a two-dimensional space where the y-axis depicts the magnitude of competitive release (CR, the number of species released from competition by the relevant releasing factor, calculated as the mean difference in the number of species between plots with and without the releasing factor), and the x-axis is the magnitude of competitive exclusion (CE, the number of species excluded by the relevant competitors in the absence of the releasing factor, calculated as the mean difference in the number of species between plots with and without the relevant competitors *in the absence of the releasing factor*). Any system for which both kinds of data are available can be represented as a single point in the CR-CE space (Fig 2A).

Systems in which the releasing factor fully compensates for competitive exclusions should fall on the line y = x (the 'compensation line', points A and B in Fig 2A) in the CE-CR space. Systems characterized by partial compensation should fall below the compensation line (points C, D, E in Fig 2A). Note that systems C and D have the same effectiveness although they differ in the magnitude of competitive exclusion, while system E shows lower effectiveness, although the magnitude of competitive release is the same as in system D. This distinction is important, and implies that variation in the effect of a releasing factor on species diversity along an environmental gradient (e.g., the commonly observed increase in the positive effect of grazing on species diversity with increasing productivity,[28, 29]) can be generated by two different mechanisms: changes in the magnitude of competitive release, and/or changes in the effectiveness of the releasing factor. We are not aware of any previous attempt to theoretically or experimentally separate these two components.

If the three basic treatments (removal of the releasing factor, removal of both the releasing factor and the competitors, and no removal) are matched within experimental blocks within the same system (as in a randomized block design), each block can be represented by a single point in the CR-CE space. In such experimental systems, the slope of a regression line fitted to the data can be interpreted as the effectiveness of the relevant releasing factor, providing that its intercept does not differ from zero (Fig 2B). Theoretically, data should fall between the lines y = 0 (no effect of the releasing factor) and y = x (full compensation), with values closer to the compensation line indicating higher effectiveness. However, real data may show deviations from this range due to noise or more complex interactions between the species (e.g., facilitation or competition among the focal species).

### A Case Study: Grazing as a Releasing Factor in a Mediterranean Annual Grassland

We use data from a field experiment that was established to investigate diversity maintaining mechanisms in annual grasslands to demonstrate our approach (see Appendix A in S1 File and [30] for a detailed description of the system). Although the experiment was not originally designed for testing the proposed framework, several properties of this system make it particularly suitable for this purpose. First, overall species richness is extremely high (>300 species), making the system particularly suitable for studying processes affecting species diversity. Second, the system is dominated by annual plants and therefore, even a short-term study is sufficient to detect demographic responses to manipulations of the competitive environment. Third, a previous study has shown that species richness in this system is strongly limited by a small number of resident grass species, which exclude a large number of forb species from the community [30]. Fourth, the system has a long history (>20 years) of cattle grazing, and previous studies have shown that grazing significantly promotes the diversity of similar systems [13, 28, 29, 31, 32]. Experiments focusing on Mediterranean grasslands also show that grazing often reduces the abundance of grasses but increases the abundance of forbs [33]. This differential response has been attributed to the taller stature of grasses in such systems, which makes them more available to the cattle [34].Thus, adopting the conceptual model presented in Fig 1A, we treated this system as one in which forbs are the focal species, grasses are competitors capable of excluding a large number of the focal species from the community, and cattle grazing is a potential releasing factor (Fig 1B).

**Fig 3:**
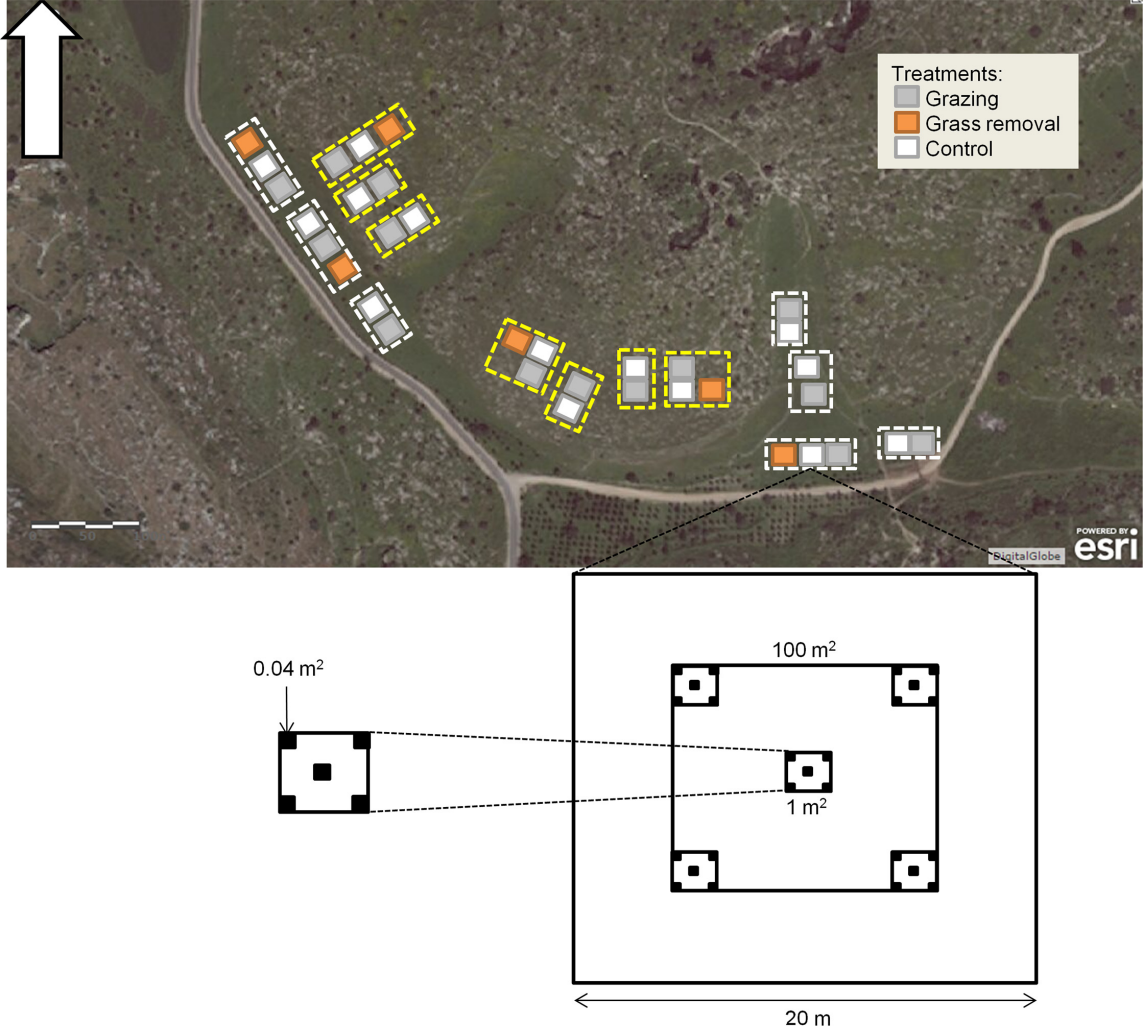
A map of the experimental system and the sampling design. Blocks located on the slopes are marked by yellow dashed lines; blocks located in the valleys are marked by white dashed lines. In each habitat there are three blocks with three treatments per block (grazing, grass removal, and control) and four blocks with two treatments (grazing and control). Each plot is 20x20m, but sampling was limited to the inner area of 10x10m. This area was sampled by 25 quadrates of 0.04m^2^ organized in five clusters of 1m^2^. Clipping experiments were conducted in the peripheral areas of control plots.

The experimental system was established in summer 2010 and consisted of three treatments: 'control' (fenced plots to prevent grazing, Fig 1C), 'grass removal' (removal of grasses within fenced plots, Fig 1D), and 'grazing' (no removal, naturally grazed plots, Fig 1B). All treatments were replicated in two types of habitats representing contrasting productivity: valleys (high-productivity habitats) and slopes (low-productivity habitats). The basic experimental unit was a plot of 20x20m, and the overall experiment included 34 plots arranged in three blocks of three plots per block (grazing, grass removal and control) and four blocks of two plots per block (grazing *vs*. control) in each type of habitat (Fig 3). Each plot was sampled in April 2012 for presence-absence of all species using 25 quadrates that were distributed hierarchically to represent three spatial scales: 0.04m^2^, 1m^2^, and 100m^2^ (Fig 3). This experimental design allowed us to measure the combined effects of competitive exclusion by grasses (d-c in Fig 1), and competitive release by grazing (b-c in Fig 1), for three scales at each of six blocks, thereby evaluating the robustness of the results to the scale at which the data are analyzed [35]. Hereafter we use the term 'quadrate' for the 0.04m^2^ scale, 'cluster' for the 1m^2^ scale, and 'plot' for the 100m^2^ scale (Fig 3).

**Fig 4:**
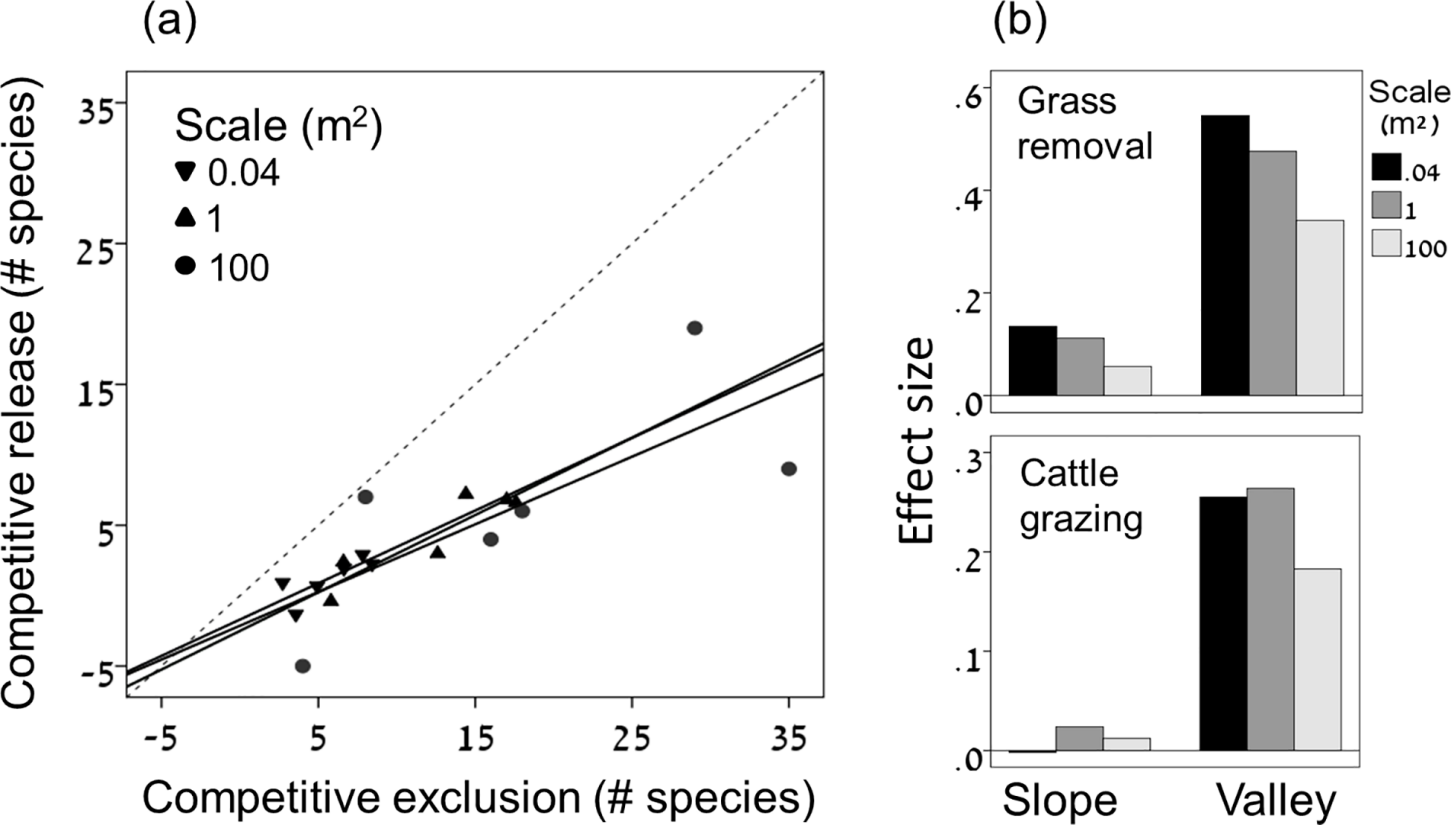
Effects of grazing and grass removal on forb richness in the study system. (a) A summary of the experimental results using the CR-CE framework. The dashed line is the compensation line (y = x). Each point represents a certain combination of habitat, block, and scale. Each line is a regression line fitted to a different scale (0.04m^2^: *R*^2^ = 0.63, *P* = 0.059; 1m^2^: *R*^2^ = 0.82, *P* = 0.012; 100m^2^: *R*^2^ = 0.54, *P* = 0.095, all slopes are significantly lower than 1 and all intercepts do not differ significantly from zero, significance levels based on standard errors of the regression coefficients). (b) Effect size (log response ratio) of the grass removal and grazing treatments under each combination of habitat (slopes *vs*. valleys) and scale (0.04, 1, and 100m^2^). Log response ratio is quantified as Log(*S*_TREATMENT_/*S*_CONTROL_), where *S* = mean number of forb species under the relevant combination of treatment, habitat and scale.

A detailed description of the results and their statistical analysis is provided in Table A and Figure A in S1 File. Here we summarize the results by plotting the data within the CR-CE framework (Fig 4A). Each point in the CR-CE space represents a certain combination of habitat, block, and scale (2 habitats x 3 blocks per habitat x 3 scales per block = 18 data points). Clearly, both competition and grazing affect the number of forb species in the study system. However, all 18 combinations fall below the line y = x, and regression lines fitted to data representing the three scales show slopes that are significantly lower than 1 and intercepts that do not differ significantly from zero in all cases (Fig 4A). These results indicate that, in spite of its considerable contribution to species richness, grazing does not fully compensate for competitive exclusion of forb species by grasses in this system.

A related analysis based on the log-response ratio as a measure of effect size (log[*S*_TREATMENT_/*S*_CONTROL_], where *S* = mean number of forb species under the relevant combination of treatment, habitat, and scale [36]) showed that the effect of grazing was much smaller than the effect of grass removal under all combinations of habitat and scale (Fig 4B). This analysis also showed that the effect of grazing on species richness was much stronger in the valleys than on the slopes (Fig 4B). This difference was consistent over all spatial scales, and was also expressed by significant interactions between the effects of grazing and habitat type on forb richness (Table A in S1 File). Two factors have contributed to this difference. First, the magnitude of competitive exclusion was consistently higher in the valleys than on the slopes (Fig 4B), making more species available for competitive release in the valley habitat. Second, for all spatial scales, the effectiveness of grazing as a releasing factor was higher in the valleys than on the slopes (mean±1S.E = 0.31±0.03 *vs*. 0.04±0.21 at the quadrate level, 0.42±0.04 *vs*. 0.18±0.13 at the cluster level, and 0.42±0.12 *vs*. 0.04±0.63 at the plot level).

**Fig 5:**
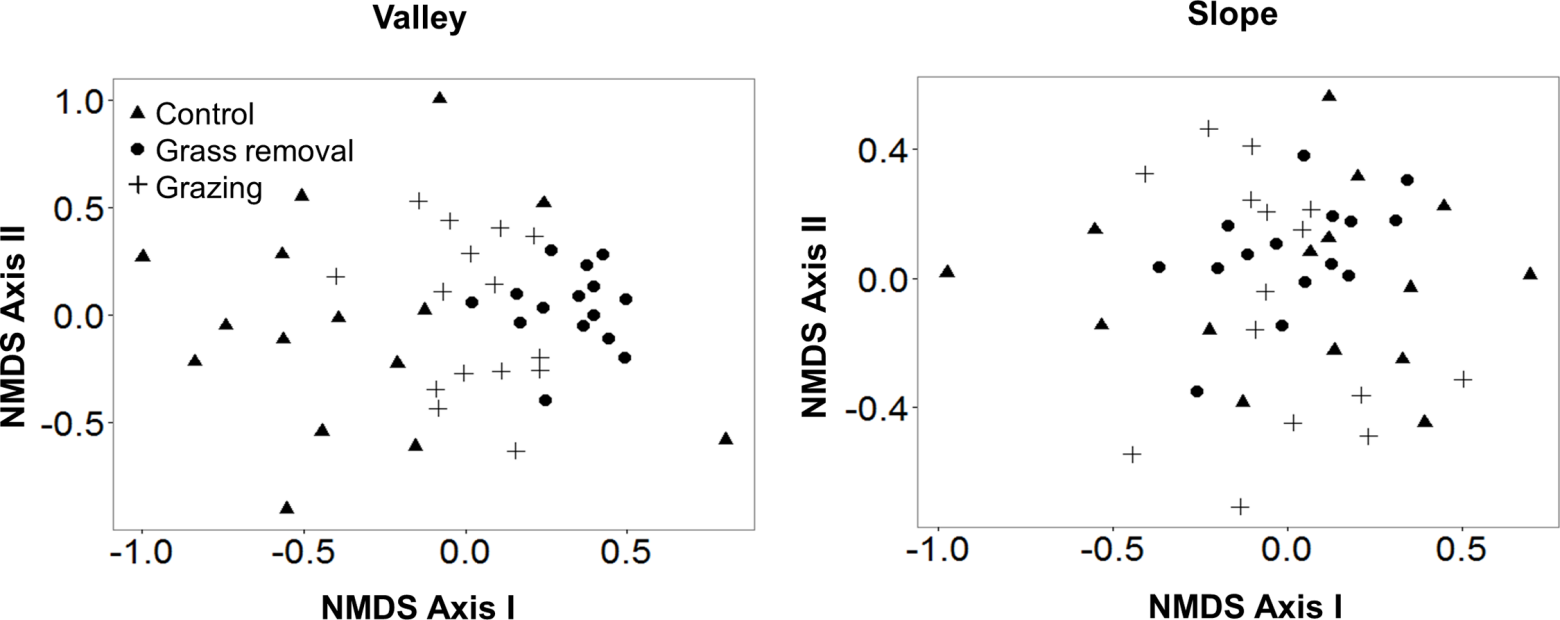
Results of ordination analysis (Nonmetric Multidimensional Scaling, NMDS) showing the effect of treatment (grass removal, grazing, and control) on forb species composition in the two habitats (valleys *vs*. slopes). Analyses were performed at the cluster scale (1m^2^) using the Jaccard index as a measure of dissimilarity.

We also performed ordination analysis using Nonmetric Multidimensional Scaling (NMDS) in order to identify the pattern of variation in forb composition among the three treatments. This analysis was performed with clusters as the observation unit, since the smaller scale (individual quadrats) often had a few species and the larger scale (plots) had a few observation units. Only clusters from blocks containing the three treatments were included in the analysis in order to have a balanced representation of the three treatments (a total of 45 observation units in each habitat). In the valleys, clusters representing the grazing treatment showed an intermediate position between those representing the control and the grass removal treatments (Fig 5), indicating that grazing partially compensates for the effect of grass competition on forb composition. On the slopes, clusters of the three treatments showed a strong overlap in their dispersion within the ordination space (Fig 5), as expected if competitive exclusion is very weak and grazing is not effective as a releasing factor.

## Discussion

Numerous empirical studies have demonstrated the ability of ecological forces such as predation, grazing, and disturbance to increase species diversity by releasing competitively inferior species from the negative effect of competition [13, 25, 31, 37–42]. Other studies have extended our theoretical understanding of the mechanisms by which such factors affect the number of species in a community [4, 6, 7, 12, 14–16]. Nevertheless, empirical data on the degree to which such 'releasing' factors are capable of offsetting species loss caused by competitive exclusion are extremely rare due to lack of appropriate experiments and methodologies. Here we present a conceptual framework that attempts to facilitate the collection, interpretation, and synthesis of such data.

### The conceptual framework

The proposed framework is based on the concept of effectiveness, which we define as the ratio between the number of species released from competitive exclusion by the releasing factor, and the number of species excluded by the relevant competitor(s) in the absence of the releasing factor. This operational definition has several advantages. The first is simplicity: it summarizes the balance between the effects of competitive exclusion and competitive release, no matter how complex they are, by a single measure ranging from zero (no release) to one (full release). Second, it provides a *standardized* measure of competitive release, thereby allowing comparison of any releasing factors under any habitat conditions. Third, it enables distinguishing between two sources of variation in the magnitude of competitive release along environmental gradients: variation due to differences in the magnitude of competitive exclusion, and variation due to differences in effectiveness of the releasing factor. Such separation is important for interpreting changes in the effects of releasing factors such as grazing and disturbance on species diversity along environmental gradients (see next section). It should also be noted that, although the proposed experimental procedure treats the releasing factor as a binary variable (with/without), the same approach can be used to compare systems representing gradients of competitive release (e.g., communities representing natural or experimental differences in the intensity of grazing).

The application of the proposed framework is not limited to systems in which the releasing factor affects only the focal species ('interaction chains' *sensu* Wootton [10], Fig 1A). Actually, in most systems, the releasing factor (predation, grazing, disturbance, etc.) affects the focal species in addition to its effect on the competitors. Such effects can be direct (e.g., by biomass removal, trampling, or seed dispersal in the case of grazing) and/or indirect (e.g., modification of habitat conditions that influence niche relationships between the component species [8]). While such effects complicate the understanding of the mechanisms generating the observed patterns, they do not influence the manner by which empirical data on competitive exclusion, competitive release, or effectiveness are interpreted.

Still, it should be emphasized that the proposed framework does not attempt to identify the *mechanisms* by which releasing factors increase the number of species in a community. In other words, the framework provides a quantitative *description*, rather than an *explanation* for the observed patterns. The actual mechanisms determining the balance between competitive exclusion and competitive release might be highly complex due to direct and indirect interactions among the competitors, the releasing factor, and the environment [5–8]. Understanding these mechanisms require auxiliary experiments that focus on specific mechanisms and are free from confounding effects (see next section for an example in our system).

Neither our conceptual framework (Fig 2), nor its empirical application, assume any kind of steady state. Rather, estimates of competitive exclusion, competitive release, and effectiveness obtained in a given experiment represent the time frame of the experiment (how much time has elapsed since the first year of manipulations), relative to the time scale of the processes by which the focal species recover from the removal of the releasing factor and the competitors (grass removal and grazing in our case study). It can be expected that experiments of different lengths in the same system would provide different estimates of competitive exclusion and competitive release, depending on the rates of these processes in the relevant system. This scale-dependency, which is a general property of any ecological experiment, does not affect the manner by which estimates of competitive exclusion, competitive release, and effectiveness are interpreted. Actually, our framework might serve as an effective tool for testing the scale-dependency of these processes by plotting the time course of competitive exclusion and competitive release as a trajectory in the CR-CE space.

It should also be noted that estimates of competitive exclusion and competitive release refer to *specific* focal species and competitors. For example, it is possible that removal of the selected competitors in a given system would lead to an increase in the number of focal species, but later, competition among the focal species themselves would reduce their number to the original (or even lower) level since new species would become dominant and competitively exclude others. Clearly, estimates of competitive exclusion obtained in such experiment do not (and need not) take into account this secondary phase of competitive exclusions. Thus, estimates of competitive exclusion and competitive release should be interpreted with respect to the particular scale *and* the particular species defined as focal species and competitors. Estimating the *overall* effect of competition on species richness in a local community is a different challenge and requires different experimental approaches (e.g., the 'community-density series' [43]).

Our analysis of the CE-CR framework (Fig 2) points to the lack of theory capable of predicting patterns of variation in the balance between competitive exclusion and competitive release. For example, while several models predict that competitive release by grazing should increase with increasing productivity [12, 15, 44], none of these models incorporates possible variation in the effectiveness of grazing as a releasing factor. Yet, as shown in Fig 2, an increase in the magnitude of competitive release can be caused by an increase in the magnitude of competitive exclusion without any change in effectiveness, by an increase in effectiveness without any change in the magnitude of competitive exclusion, or by a combination of both mechanisms (as in our case study, see below). Currently no theory is capable of providing predictions concerning these alternative scenarios.

### Interpretation of the case study

As expected, cattle grazing significantly increased species richness in our study system (Fig 4B, Figure A in S1 File). This finding is consistent with previous studies focusing on Mediterranean grasslands [28, 29, 45]. Several lines of evidence suggest that the mechanism underlying this effect was competitive release of forb species from the negative effect of grasses. First, in the absence of grazing, the number of forb species was strongly limited by the presence of grasses (Fig 4B). Second, the positive effect of grazing on species richness was entirely due to an increase in the number of forb species, with no effect on grasses (Figure A in S1 File). Third, when patterns of forb composition were analyzed using ordination analysis, plots representing the grazing treatment occupied an intermediate position between the grass removal and control treatments, indicating that grazing mitigates the competitive effects of grasses on forb composition (Fig 5). All of these findings are consistent with the hypothesis that grazing promotes the diversity of the study communities by releasing forb species from the competitive effect of grasses (see also [26]).

**Fig 6:**
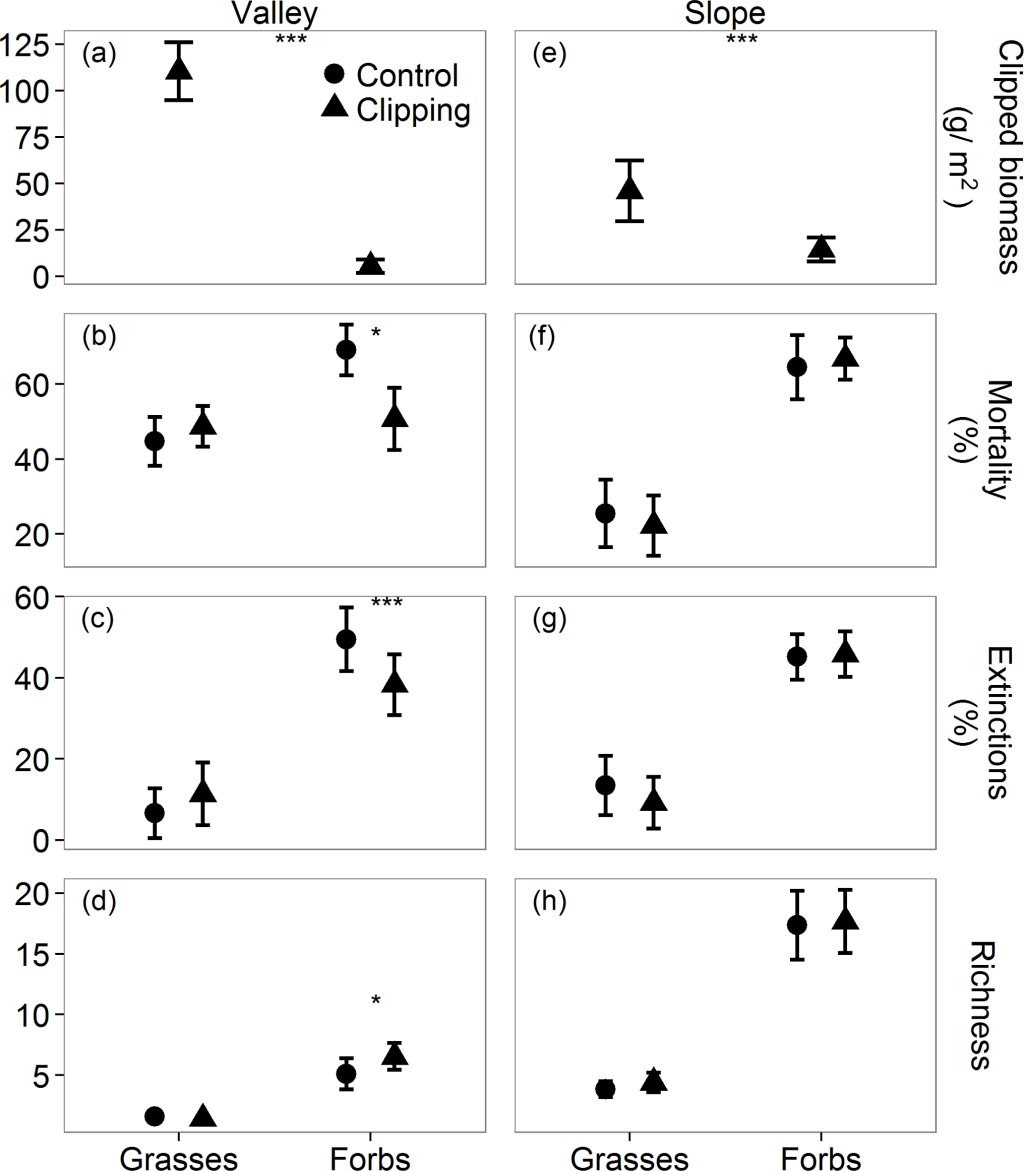
Results of a small-scale clipping experiment mimicking the effect of biomass removal by the cattle on the vegetation in the study area (removal of all shoots higher than 7 cm). The experiment was conducted within experimental units of 0.16m^2^ protected from grazing (see Appendix B in S1 File for details). (a, e) Clipped biomass of forbs and grasses in the two habitats. (b, f) Seedling mortality. (c, g) Extinction rates. (d, h) Species richness at the end of the growing season. Bars represent 95% confidence levels. Significant differences between clipped biomass of grasses and forbs (a, c) and between clipping treatments (b-d, f-h) are marked by asterisks.

To better understand the mechanisms by which grazing affects species diversity in the study communities we conducted auxiliary clipping experiments that were designed to mimic biomass removal by the cattle (removal of all shoots higher than 7cm, see Appendix B in S1 File). These small-scale experiments were conducted within fenced plots, thereby controlling for other potentially confounding effects of the cattle (trampling, fertilization, seed dispersal, etc.). The responses of both forbs and grasses to clipping were very similar to their responses to grazing (Fig 6): grasses suffered a much higher reduction in biomass than forbs due to their taller stature, but only forbs showed a significant increase in species richness (see Figures A and B in S1 File for the effect of grazing on richness and biomass, respectively). The lack of a significant effect of grazing on forb richness in the slope habitat (Figure A in S1 File) is also consistent with the observed responses to clipping (Fig 6). These findings strengthen support for our hypothesis that the main mechanism by which grazing promotes the diversity of forb species in the study system is removal of grass biomass.

Nevertheless, our data demonstrate that the positive effect of grazing on forb richness was too weak to counterbalance the negative effect of competitive exclusions. This limited capability is expressed by the fact that the effectiveness of grazing was lower than 1 regardless of habitat type (slopes or valleys), spatial scale (0.04, 1, or 100 m^2^) or method of calculation (CR-CE ratio or regression slope). Even in the valleys, where grazing had a remarkable effect on forb richness, competition with grasses reduced the number of forb species to 32–46% of its potential (defined as the number of species in grass removal plots), while grazing raised the number of forb species to only 49–64% of its potential. These results indicate that, despite its ability to significantly promote species richness, grazing does not fully compensate for species loss caused by competitive exclusion in this system.

It should also be noted that the significant positive effect of grazing on species richness was limited to the valley habitat, and on the slopes its effect was insignificant at all scales (Table A and Figure A in S1 File). This finding resembles results from previous experiments in the region [28], and is attributed to the higher productivity of the valleys relative to the slopes (Figure B in S1 File). Higher productivity promotes light competition, which is the main cause of competitive exclusion in grassland communities [18, 46, 47]. Grazing releases species from light competition [25], and therefore it can be expected to have a stronger positive effect on species richness under high levels of productivity[13]. Our previous observation that competitive exclusion of forb species is much more frequent in the valleys than on the slopes [30] is consistent with this explanation.

An important insight emerging from our conceptual framework is that differences in the effect of grazing on species richness may originate from two distinct mechanisms: underlying differences in the magnitude of competitive exclusion; and/or differences in the effectiveness of grazing as a releasing factor. Distinguishing between the two mechanisms is crucial for interpreting observed patterns of variation in the effect of grazing on species richness along environmental gradients. The first mechanism should be considered a null model in such studies, since it only affects the species pool that is available for competitive release by the herbivores (if more species are excluded, competitive release might be higher simply due to a larger species pool). The second mechanism implies true differences in the effect of grazing as a releasing factor, and may reflect underlying differences in the density of the herbivores, and/or differences in the per-capita ‘efficiency’ of competitive release (how many species an average herbivore releases from competition). Clearly, any attempt to understand such differences and their underlying mechanisms requires quantification of the magnitudes of competitive exclusion, competitive release, and effectiveness.

Our analysis of effect size indicates that differences in the magnitude of competitive release between the two habitats were consistently larger than the corresponding differences in the magnitude of competitive exclusion (Fig 4B). This result implies that, in addition to the differences in the magnitude of competitive exclusion, the two habitats differed also in the effectiveness of grazing as a releasing factor. Indeed, our results indicate that the average effectiveness of grazing was consistently higher in the valley habitat under all spatial scales. As far as we are aware, this is the first time that observed differences in the effect of grazing on species diversity are decomposed into these two components.

### Summary and conclusions

Competitive exclusion and competitive release are important determinants of species diversity. Research in community ecology has provided much evidence for competitive exclusion and competitive release in natural communities, but studies in which both processes have been quantified in the same system are extremely rare. Here we argue that experimentally quantifying both processes is crucial for understanding observed patterns of variation in the effect of releasing factors such as predation, grazing and disturbance on species diversity; and propose a simple though general framework for the design and interpretation of such experiments. We believe that applying the proposed framework in a wide spectrum of communities and ecosystems is feasible, and may fill a major gap in current research in community ecology.

## Supporting Information

S1 Dataset Biodiversity grazing-grass removal experiment. (CSV)

S2 Dataset Biomass grazing experiment. (CSV)

S3 Dataset Biodiversity clipping experiment. (CSV)

S4 Dataset Biomass clipping experiment. (CSV)

S1 File A detailed description of the methods, this file includes the experimental design, supporting figures, supporting tables and description of all the supporting datasets. (DOCX)
