# Supplementary material for: Quantifying Competitive Exclusion and Competitive Release in Ecological Communities: A Conceptual Framework and a Case Study
Source: PLoS One. 2016 Aug 18;11(8):e0160798. doi: 10.1371/journal.pone.0160798 (PMC4990188; doi:10.1371/journal.pone.0160798)
Supplement: S1 File — (DOCX) [file pone.0160798.s005.docx]

**SUPPORTING INFORMATION**

**Quantifying Competitive Exclusion and Competitive Release in Ecological Communities: A Conceptual Framework and a Case Study**

**Table of contents**

**APPENDIX A.** Description of the experimental system

# APPENDIX B. The clipping experiment

**Table A.** Effect of grazing, habitat type and their interaction on forb richness, grass richness, and total richness

**Fig. A**. Effect of grazing on forb richness, grass richness, and total richness

**Fig. B.** Effect of grazing on dry biomass of forbs and grasses

**APPENDIX C:** descriptions of the csv data files

**APPENDIX A. Description of the experimental system**

**The study site**

The experimental system is located in Beit-Govrin National Park, Israel. There are two main types of habitats in this area: valleys, characterized by deep (>100cm) alluvium soil, and slopes, characterized by shallow (usually <50cm) rendzina soil. The difference in soil depth results in a considerable difference in annual productivity which is much higher in the valleys. The vegetation in both habitats is dominated by annual species, with *Hordeum spontaneum* being the dominant species in the valleys and *Stipa capensis* dominating the slopes. The area is subject to cattle grazing during the winter, and in wet years, also in the early summer. A grazing phase takes 2-3 weeks, during which grazing pressure is moderate (3.5 ha/cow). There are no consistent differences in grazing pressure between the two types of habitats although occasionally one habitat might be grazed more heavily than the other.

**Experimental design**

In summer 2010, six blocks, each containing three plots of 20x20m, were marked in the study area. One half of the blocks were located in valleys and the other half on slopes. One plot in each block was fenced to serve as an experimental control (grazing removal, Fig. 1c). A second plot was fenced for the purpose of the grass removal treatment (removal of both grazing and grasses, Fig. 1d). Grasses were removed from these plots following germination using the herbicide FOCUS ULTRA (cycloxidim 10%), a selective post-emergence herbicide that does not affect broadleaf species. The third plot in each block was left unfenced to serve as a control (no removal, Fig. 1b). This experimental design allowed us to measure the combined effects of competitive exclusion by grasses (d-c in Fig. 1), and competitive release by grazing (b-c in Fig. 1), in six independent blocks. Note that we treat the fenced plots with grasses present (d in Fig. 1) as experimental control in this design.

Four additional blocks, each containing a matched pair of grazed *vs*. ungrazed (fenced) plots were established in each type of habitat in order to increase sample size of the grazing treatment because grazing was expected to have a weaker and less homogeneous effect on species diversity than grass removal. Thus, the overall experimental system included 34 plots arranged in three blocks of three plots per block (grazing, grass removal and control) and four blocks of two plots per block (grazing *vs*. control) in each type of habitat (Fig. 3).

All plots were sampled in April 2012 (end of the second year of the experiment) using the same standard protocol. Species presence-absence data were determined in 25 quadrates of 20x20cm (0.04m^2^) within the central 10x10m area of each plot (Fig. 3). The sampling quadrates were aggregated in clusters of five quadrates per cluster, with each cluster representing an area of 1x1m (Fig. 3). The five clusters were located at the four corners of the central 10x10m area and in its center (Fig. 3). This sampling design allowed us to evaluate the robustness of our results to the scale at which the data were analyzed.

**Statistical analysis**

The effect of grazing on species richness was determined at three hierarchical scales within each habitat: individual quadrates (0.04m^2^), clusters of quadrates (1m^2^), and plots (100m^2^). Richness of clusters and plots was determined by aggregating the data from the relevant quadrates. At a first step we tested the effect of grazing removal, habitat type, and their interaction on richness at the quadrate scale using a linear regression model. The results showed that residuals of quadrates within the same cluster were strongly and significantly correlated (Moran's I = 0.258, P < 0.001), those of quadrates located at different clusters within the same plot were weakly correlated (Moran's I = 0.024, P = 0.002), and those of quadrates located at different plots were uncorrelated. In order to account for the spatial dependency between quadrates within the same cluster, the quadrate-scale (0.04m^2^) richness was averaged for each cluster, and the analysis was repeated using Simultaneous Autoregressive Regression (SAR) model with clusters as the basic unit of the analysis:

(1) $y_{ijk}=\beta_{0}+\beta_{1}Habitat_{i}+\beta_{2}Treatment_{j}+\beta_{3}(Habitat\times Treatment)_{ij}+\varepsilon_{ijk}$, $\varepsilon=\lambda W\varepsilon+\xi$

where *y* is the average richness per quadrate within a given cluster, *i* is an index for a pair of matched plots in a given habitat (valley = 0, slope = 1), *j* is an index for a particular plot in a given habitat and under a given treatment (control = 0, grazing removal = 1), *k* is an index for the particular cluster of quadrates, $\varepsilon$ is the vector of error terms, spatially weighted using the weights matrix $W$(we used standardized weights where each neighbor in the plot-pair has the same weight), $\lambda$ is the spatial error coefficient, and $\xi$ is a vector of uncorrelated error terms. For the cluster scale (1m^2^), a similar SAR model was used, but without averaging, i.e., with $y_{ijk}$representing the number of species per cluster. For the plot scale (100m^2^), we used a linear regression model similar to the spatial model, but with no correlation between the error terms because no spatial autocorrelation was detected at that scale:

(2) $y_{ij}=\beta_{0}+\beta_{1}Habitat_{i}+\beta_{2}Treatment_{j}+\beta_{3}(Habitat\times Treatment)_{ij}+\varepsilon_{ij}$, $\varepsilon_{ij}\sim N(0,\sigma_{\varepsilon}^{2})$.

A similar analytical procedure was used to analyze the effect of grass removal on species diversity (see Segre *et al.* 2014 for details).

# APPENDIX B. The clipping experiment

# The clipping experiment was conducted within fenced plots and included 20 pairs of 0.16m^2^ quadrates in the valleys and 28 paired quadrates on the slopes. In each pair, one quadrate was left as a control and the other was clipped during the grazing period of the second year of the experiment by removing all shoot parts higher than 7cm (based on observations in grazed areas). The clipped biomass was dried for 72 hours at 60^0^ C, separated to grass and forb species, and weighed. All quadrates were sampled twice: following germination (before the clipping treatment) and at the end of the growing season. All individuals of all species were counted in each census. These data were used to determine dry weight of the clipped biomass (Fig. 6 a, e), mortality rates (percentage of individuals that germinated but did not survive to the flowering stage, Fig. 6 b, f), extinction rates (percentage of species that germinated but did not survive to the flowering stage, Fig. 6 c, g), and species richness (the number of species survived to the flowering stage, Fig. 6 d, h) of forb *vs*. grass species at each quadrate. The clipped biomass was analyzed using repeated measures analysis of variance with forbs *vs*. grasses as a within-subject factor and habitat type (valleys *vs*. slopes) as a between-subject factor. The biomass data were log-transformed prior to the analysis. The effects of clipping on mortality rates, extinction rates, and species richness, were analyzed using paired t-tests. Separate tests were performed for grasses and forbs in each type of habitat.

# Table A. Effect of grazing, habitat type and their interaction on forb richness, grass richness, and total richness, at three spatial scales. The two smaller scales (0.04 and 1m^2^) were analyzed using Simultaneous Autoregressive Regression (SAR) models and the largest Scale (100m^2^) was analyzed using linear regression model (see text for statistical details). Significant results are shown in bold. The simultaneous autoregressive error coefficient Lambda is reported for SAR models with significance level based on a Likelihood Ratio (LR) test for spatial autocorrelation.

| **Group** | **Effect** | **0.04m^2^** | |  | **1m^2^** | |  | **100m^2^** | |
| --- | --- | --- | --- | --- | --- | --- | --- | --- | --- |
|  |  | ***Z*** | ***P*** |  | ***Z*** | ***P*** |  | ***t_8_*** | ***P*** |
| **Forbs** | **Grazing** | **3.81** | **< 0.001** |  | **4.17** | **< 0.001** |  | **2.62** | **0.015** |
|  | **Habitat** | **11.24** | **< 0.001** |  | **13.09** | **< 0.001** |  | **12.24** | **< 0.001** |
|  | **Grazing x Habitat** | **-2.34** | **0.019** |  | **-2.84** | **0.045** |  | **-2.07** | **0.049** |
|  | **Lambda^*^** | **LR = 6.23** | **0.013** |  | **LR = 5.01** | **0.025** |  |  |  |
| **Grasses** | **Grazing** | -0.68 | 0.498 |  | 0.18 | 0.855 |  | 1.03 | 0.311 |
|  | **Habitat** | **6.38** | **< 0.001** |  | **8.82** | **< 0.001** |  | **10.76** | **<0.001** |
|  | **Grazing x Habitat** | 1.38 | 0.167 |  | 1.49 | 0.137 |  | 0.73 | 0.471 |
|  | **Lambda^*^** | LR = 1.54 | 0.214 |  | LR = 3.34 | 0.068 |  |  |  |
| **Total** | **Grazing** | **3.44** | **< 0.001** |  | **3.90** | **< 0.001** |  | **2.80** | **0.010** |
|  | **Habitat** | **12.80** | **< 0.001** |  | **15.05** | **< 0.001** |  | **14.16** | **<0.001** |
|  | **Grazing x Habitat** | -1.90 | 0.057 |  | **-2.28** | **0.023** |  | -1.92 | 0.067 |
|  | **Lambda^*^** | LR = 3.36 | 0.067 |  | LR = 2.58 | 0.108 |  |  |  |

**Fig. A**. Effect of grazing on forb richness, grass richness, and total richness, in the two types of habitats (valleys and slopes) and at the three spatial scales (0.04m^2^, 1m^2^, and 100m^2^). Bars represent 95% confidence levels, significant effects are marked by asterisk (*P<0.05, **P<0.01, ***P<0.001). A separate analysis was performed for each scale in each habitat.

**Fig. B.** Effect of grazing on dry biomass of forbs and grasses in the two types of habitats (valleys and slopes). Bars represent 95% confidence levels, significant effects are marked by asterisk (*P<0.05, **P<0.01, ***P<0.001). Biomass was sampled in the peripheral areas of fenced *vs*. unfenced plots.


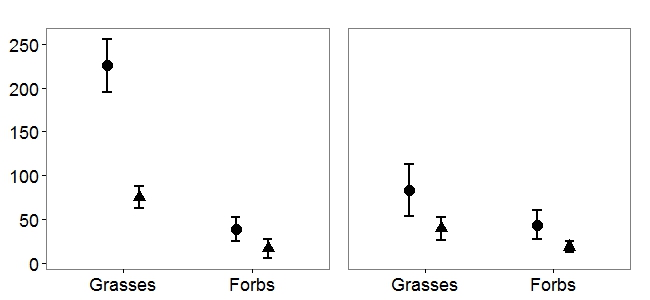


**Biomass (g/m^2^)**

Control

Grazing


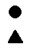


**Valley**

**Slope**

*******

*******

*******

*******

**APPENDIX C: Metadata for csv datafiles**

File B: "biodiversity grazing-grassremoval experiment.csv"

Description: Species presence-absence data collected in the grazing, grass removal and control plots

| Column | Definition |
| --- | --- |
| Sampling_Year | Year in which the sampling was conducted |
| ID Sample | sampled quadrate running unique id |
| Block | block no. for a set of paired treatments (grazing + control / grazing + control + grassremoval) |
| Fence | fence no. for identification (treatment was applied at the fence scale) |
| Fiver | a fiver aggregates 5 neighbouring samples in the fence and is used to calculate richness at the 1m scale (see sampling design in the original paper) |
| Quadrate | a single sampling unit. The presence counts were conducted at the quadrate scale. |
| Habitat | where the block is located (valley/slope) |
| Treatment | what treatment did the fence receive (grazing / grass removal / control) |
| IsraelTM-X | x-ccordinate of the sample quadrate in Israel-TM grid (meters) |
| IsraelTM-Y | y-ccordinate of the sample quadrate in Israel-TM grid (meters) |
| Lat_Name | Latin name of the observed species |

File S3: "biomass grazing experiment.csv"

Description: Dry-biomass data collected in the grazing, grass removal and control plots

| Column name | Definition |
| --- | --- |
| Block | Block no. for a block of paired fences with different treatments |
| Plot | Fence no. treatment were applied at fence level |
| Habitat | location of block (slope / valley) |
| Treatment | Treatment assigned to plot |
| Sample | 1-7 samples were taken in each plot |
| Grasses_g | dry-weight of grasses in grams |
| Forbs_g | dry-weight of forbs in grams |
| Unidentified_g | dry-weight of unidentified species in grams |

File S4: "biodiversity clipping experiment.csv"

Description: Abundance of seedlings and adults in clipping plots

| Column name | Definition |
| --- | --- |
| Fence | Fence no.for identification |
| Quadrate | code for the treatment quadrate for identification (unique in the fence) |
| Habitat | location of plot (slope / valley) |
| Clipping | level of clipping treatment: 0-no clipping (control), 2-clipped twice (clipping) |
| count_type | describes the origin of abundance data: if from seedlings count (winter) or adults count (spring) |
| Lat_Name | Species name (latin) |
| sum | abundance of species in the quadrate in the relevant count (seedlings/adults) |

File S5: "biomass clipping experiment.csv"

Description: Clipped biomass from the clipping plots

| Column name | Definition |
| --- | --- |
| Fence | Fence no.for identification |
| Quadrate | code for the treatment quadrate for identification (unique in the fence) |
| Habitat | location of block (slope / valley) |
| Type | type of biomass (forbs/grasses) |
| Clipping_1_g | biomass in first clipping of the season in grams |
| Clipping_2_g | biomass in second clipping of the season in grams |
| total_g | total clipped biomass during the season in grams |
| total_g_per_m | total clipped biomass during the season calculated in grams per m2 |
